# A Case Report of a High-Altitude Acute Pulmonary Embolism (HA-PE): A Catastrophic Masquerader of High-Altitude Pulmonary Edema (HAPE)

**DOI:** 10.7759/cureus.40975

**Published:** 2023-06-26

**Authors:** Keyur Saboo, Varun Daiya, Sourya Acharya, Sunil Kumar, Samarth Shukla

**Affiliations:** 1 Internal Medicine, Jawaharlal Nehru Medical College, Wardha, IND; 2 Department of Medicine, Jawaharlal Nehru Medical College, Wardha, IND; 3 Department of Medicine, Jawaharlal Nehru Medical College, Datta Meghe Institute of Medical Sciences, Wardha, IND; 4 Department of Pathology, Jawaharlal Nehru Medical College, Datta Meghe Institute of Medical Sciences, Wardha, IND

**Keywords:** catheter-directed therapy, case report, thrombolysis, breathlessness, high altitude

## Abstract

Pulmonary embolism is a life-threatening emergency and, if not identified and treated, can cause catastrophic consequences. The most common cause of pulmonary embolism is deep vein thrombosis (DVT). There are established criteria to diagnose pulmonary embolism. One of the rare causes of pulmonary embolism is exposure to high altitude (HA). We present a case of a 51-year-old male without any co-morbidities, who, after traveling to an HA destination, developed acute onset dyspnea and was labeled as a case of HA pulmonary edema (HAPE). Further investigations in our hospital revealed a massive pulmonary embolism. Post thrombolysis, the patient was comfortable. After 48 hours, the patient started to walk at a normal pace without any symptoms and was discharged after seven days. This case report emphasizes the importance of keeping rare possibilities, such as pulmonary embolism, in such rare scenarios.

## Introduction

High altitude (HA) is usually defined as elevations or heights above 2,700 m/9,000 feet, and extreme HA is elevation/heights above 5,500-5,800 m/18,000-19,000 feet. The physiological complications that occur after ascending to HAs are acute mountain sickness, HA cerebral edema, and rarely HA pulmonary edema (HAPE) [1,2]. The possible pathophysiology behind these complications is hypobaric hypoxia, leading to increased perfusion of capillaries causing increased pulmonary capillary pressure and pulmonary edema. Hypoxia also has critical interaction with the coagulation cascade, leading to a hypercoagulable state and predisposition for developmental thrombosis. A study has shown that low landers rapidly ascending to such HAs can develop pulmonary embolisms even if they do not have any comorbidities [3,4,5]. A state of hyperfibrinogenemia persists till the person remains in HAs, and it regresses after descent in a few weeks; a prolonged stay, usually more than five months, in HAs predisposes for the formation of deep vein thrombosis (DVT) [6,7,8]. The role of coagulation cascade causing HA pulmonary embolism (HA-PE) has created specific interest in various scientific studies, and activation of the components of Virchow's triad, such as endothelial injury, stasis of venous blood, and hypercoagulability, all of which has been implicated as triggering events in HA-PE. We present a rare case of an HA-induced pulmonary embolism that developed acutely in the absence of significant risk factors, possibly as a result of an acutely developing hypercoagulable state that progressed within the span of seven days, which made the patient seek emergency care in our hospital.

## Case presentation

A 51-year-old male patient came to our hospital with complaints of breathlessness on mild exertion for three days. Seven days before, he went to an HA on a pilgrimage to a religious place, which is about 13,000 feet above ground level. There was no history of prolonged immobilization. The ascent was relatively rapid. After two days, he developed mild headaches and muscle cramps, which were relieved by the tablet paracetamol. On the third day, while climbing a specific destination on foot, he developed breathlessness and extreme fatigue. He then informed his ailments to fellow travelers and was advised not to climb. He was taken to the local medical clinic where he was treated as a case of acute mountain sickness. On asking leading questions, he revealed that, for approximately four to five hours, he was on nasal oxygen and received IV fluids after that he felt better and was advised to come back. He still had exertional dyspnoea NYHA grade 2, which progressed to NYHA grade 3 at the time of presentation.

On examination in our hospital, his pulse was 110 beats/min regular, blood pressure was 96/50 mm of Hg in the right arm, respiratory rate was 18 cycles/min, and SpO2 was 93% while breathing ambient air. JVP was raised to 11 cm of H2O at a 45-degree angle. When asked to walk 6 m, the saturation fell to 88%. His respiratory system examination did not reveal any abnormality. His cardiovascular system examination revealed tachycardia, loud s1, normal p2, no s3,s4, or murmur. The patient was immediately admitted to the intensive care unit (ICU) with the possibility of acute cor pulmonale.

**Figure 1:**
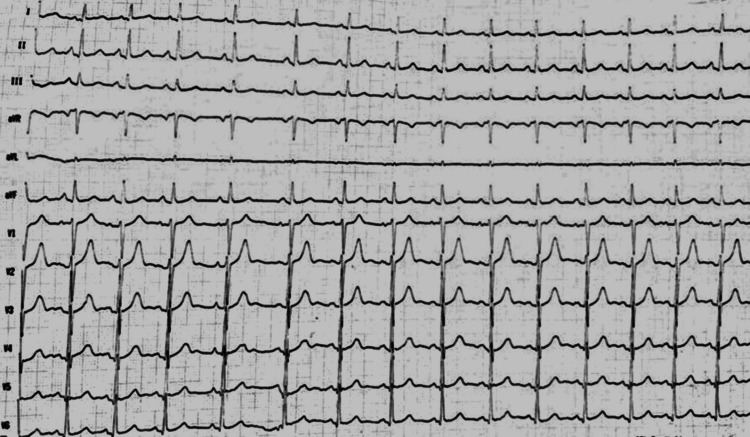
ECG showing sinus tachycardia

**Figure 2:**
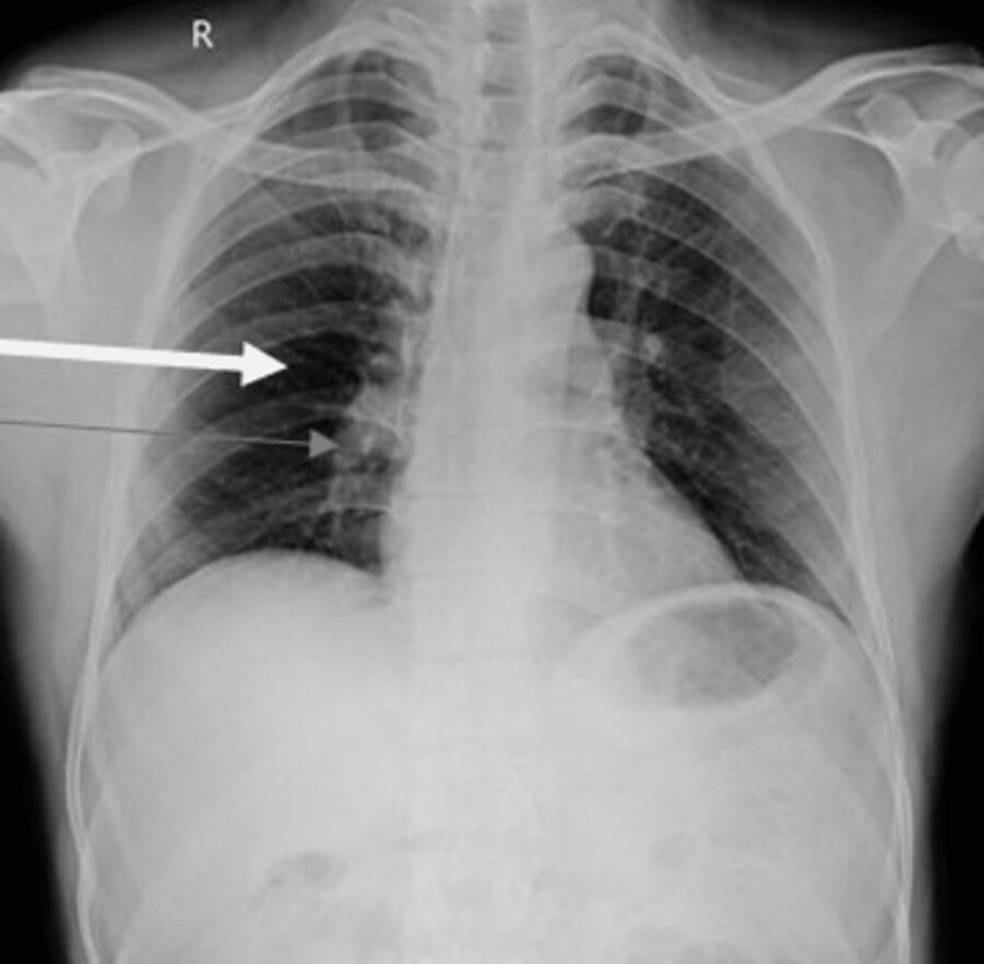
Chest X-ray revealed classical X-ray findings of pulmonary embolism The white arrow indicates focal oligemia (Westermark sign), and the grey arrow shows the prominent right descending pulmonary artery enlargement (Palla’s sign).

Investigations revealed: hemoglobin = 10.2 g/dl, total leucocyte count = 5,600/cu.mm, total platelet count = 1.2 lacs/cu.mm, activated partial thromboplastin clotting time = 29.8 seconds, prothrombin time = 12.0 seconds, and INR = 1.00 ng/ml. Serum creatinine, serum sodium, serum potassium, SGPT, alkaline phosphatase, and total bilirubin were within normal limits. Quantitative D-dimer was raised to 1122 ng/ml (> 500 suggests pulmonary embolism), and NT-Pro BNP was 204 pg/ml (normal range: less than 400 pg/ml). Another thrombophilia profile was within normal limits. ECG showed sinus tachycardia (Figure 1). Chest X-ray revealed classical X-ray findings of pulmonary embolism (Figure 2). 2D echocardiography was suggestive of right ventricular dilatation as a significant finding. The rest of the findings were within normal limits.

**Figure 3:**
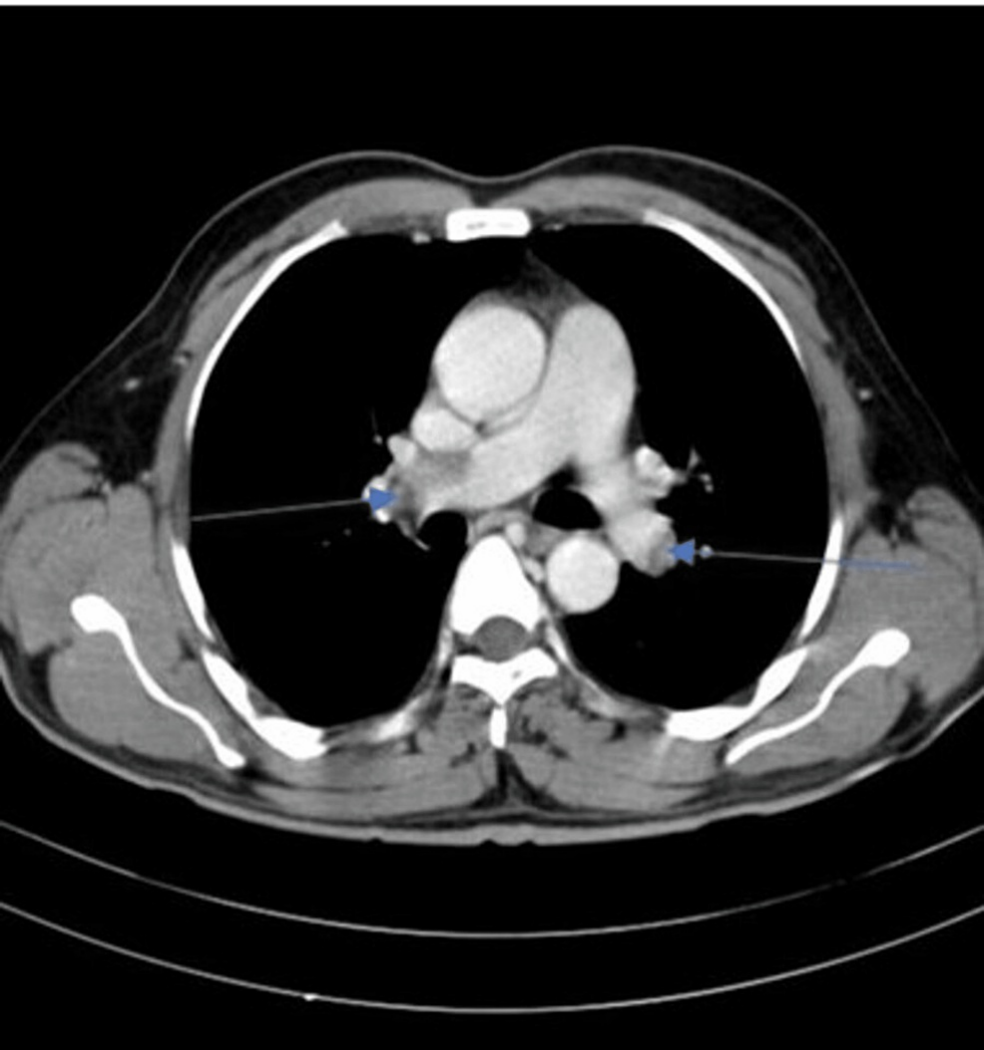
Axial image of computed tomography (CT) pulmonary angiogram, showing a filling defect in the right pulmonary artery (arrow) and left pulmonary artery (arrow)

CT pulmonary angiography documented a thrombus in the right main pulmonary artery and its segmental branches and a thrombus in the left pulmonary artery and its segmental branches (Figure 3). Doppler studies of lower limbs showed normal deep veins in both lower limbs.

**Figure 4:**
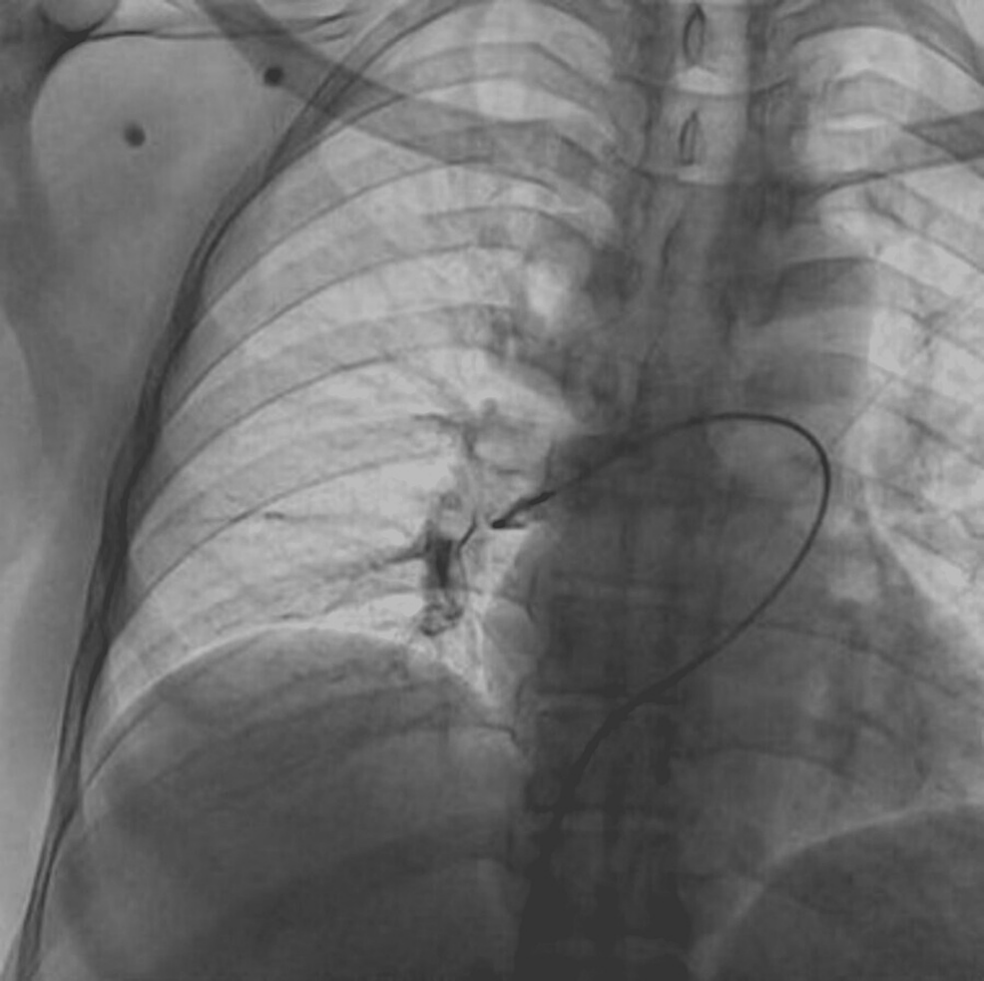
The right femoral vein was canulated, and the catheter was transcended up to the pulmonary artery where thrombolysis was done using alteplase

Intra-arterial thrombolysis was performed with Alteplase 20 mg due to decreased saturation and hypotension (SpO2 was 80% on room air, and blood pressure was 80 systolic) (Figure 4 and Video 1). A study has shown that intraarterial thrombolysis is superior to intravenous thrombolysis in patients with high-risk pulmonary embolisms [9]. The patient was then transferred to the ICU, and heparin infusion commenced for 48 hours. After 48 hours, heparin infusion was overlapped with tablet apixaban 5 mg od. Apixaban was suggested to continue for six months, and the patient was advised to check up after two months. At the time of the follow-up, there was no dyspnoea, no clinical symptoms, and saturation on room air was 100%.

**Video 1:** Video showing thrombolysis in which the right femoral vein was canulated and the catheter was transcended up to the pulmonary artery where thrombolysis was done using alteplase

## Discussion

HAs have a significant impact on the body's coagulation cascade. Numerous risk factors for thrombosis exist for climbers who spend weeks at high elevations. When starting HA, there is a temporary hypercoagulable state that lasts for a few weeks, but gradually regresses as the patient becomes used to it. This results from platelet dysfunction and a brief rise in clotting factors. Numerous case reports of venous thromboembolism among HA mountaineers have sparked interest in the field of science regarding the function of coagulation in HA diseases. Ascent to HAs results in hypobaric hypoxia, which is known to affect human physiology by reducing tissue oxygenation and causing other sympathetic compensatory alterations, such as raised systemic blood pressure, arrhythmias, and vasoconstriction.

Individuals who developed HA pulmonary hypertension had higher levels of plasma fibrinogen, platelet adhesiveness, platelet factor III, factor V, and factor VIII, according to Singh and Chauhan [10]. According to some research, intravascular stimulation of the coagulation process is also thought to be a contributing factor to pulmonary hypertension at HAs [11].

The link between hypoxia and hemostasis has been the subject of numerous research endeavors. It is hypothesized that a protracted stay at HAs could cause the coagulation system to become activated due to an increase in hematocrit and blood viscosity [12,13,14,15,16]. During a simulated HA exposure, Maher and colleagues looked at a number of coagulation and platelet aggregation parameters and discovered that several parameters had changed, which is indicative of coagulopathy [13].

In a classic study, Kotwal and colleagues conducted a prospective cohort study at a height of 3,500 m above sea level and came to the realization that the combination of erythrocytosis, increased platelet count, platelet activation, and raised fibrinogen level, combined with hypoxia and dehydration at HA, cause a thrombotic milieu to occur, leading to thrombosis in healthy people or in asymptomatic cases with inherited/acquired thrombophilia [15].

## Conclusions

This case report demonstrates an elevated risk of hypercoagulability at HAs that may cause DVT or pulmonary embolism. Compared to lowlanders, fatal pulmonary embolism cases are allegedly highly common among visitors to HAs. Future research should take this clinical paradox into account. Studies in this area are currently largely undefined. Larger subject populations and more INR assessments will result in better and more conclusive findings. For more accurate changes in coagulation parameters, an altitude range might be studied. To prevent loss of life, newer, more dependable early diagnostic techniques and prevention tactics must be developed. Warfarin may not always be necessary; other therapies may be used instead. Understanding the elevated risk of thrombo-embolic disorders at HAs as well as the potential underlying processes will require more research.

## References

[1] Swenson ER, Maggiorini M, Mongovin S, Gibbs JS, Greve I, Mairbäurl H, Bärtsch P (2002). Pathogenesis of high-altitude pulmonary edema: Inflammation is not an etiologic factor. JAMA.

[2] West JB, Colice GL, Lee YJ (1995). Pathogenesis of high-altitude pulmonary oedema: Direct evidence of stress failure of pulmonary capillaries. Eur Respir J.

[3] Genton E, Ross AM, Takeda YA, Vogel JH (1970). Alterations in blood coagulation at high altitude. Hypoxia, High Altitude and the Heart: 1st Conference on Cardiovascular Disease, Snowmass-at-Aspen, Aspen, Colo., January 1970.

[4] Dilly PN (1976). Mountain medicine. A clinical study of cold and high altitude. Michael Ward. 140 × 215 mm. Pp. 376 + x, with 21 illustrations. 1975. London: Crosby Lockwood Staples. £10. Br J Surg.

[5] Hussain T, Niaz A (2002). Deep venous thrombosis at high altitude. J Pak Med Assoc.

[6] Virchow R (1856). GesammelteAbhandlungenzurWissenschaftlichen Medicine. https://wellcomecollection.org/works/m3tp5x6w/items?canvas=2.

[7] Jha SK, Anand AC, Sharma V, Kumar N, Adya CM (2002). Stroke at high altitude: Indian experience. High Alt Med Biol.

[8] Cucinell SA, Pitts CM (1987). Thrombosis at mountain altitudes. Aviat Space Environ Med.

[9] Brown KN, Devarapally SR, Lee LS, Gupta N (2023). Catheter-Directed Thrombolysis of Pulmonary Embolism. https://www.ncbi.nlm.nih.gov/books/NBK536918/.

[10] Singh I, Chohan IS (1972). Blood coagulation changes at high altitude predisposing to pulmonary hypertension. Br Heart J.

[11] Ulloa NA, Cook J (2023). Altitude-Induced Pulmonary Hypertension. https://www.ncbi.nlm.nih.gov/books/NBK555925/.

[12] Maher JT, Levine PH, Cymerman A (1976). Human coagulation abnormalities during acute exposure to hypobaric hypoxia. J Appl Physiol.

[13] Hudson JG, Bowen AL, Navia P, Rios-Dalenz J, Pollard AJ, Williams D, Heath D (1999). The effect of high altitude on platelet counts, thrombopoietin and erythropoietin levels in young Bolivian airmen visiting the Andes. Int J Biometeorol.

[14] Charan Bagga, Iftekhar Ansari, Amrutha Garikapathi, Irshadvs Irshadvs, Srinivas Naik, Sachin Agrawal, Sunil Kumar (2020). Status epilepticuspresenting feature of pulmonary embolism: Rarest of rare combination. Medical Science.

[15] Kotwal J, Chopra GS, Sharma YV, Kotwal A, Jatin B (2004). Study of pathogenesis of thrombosis at high altitude. Indian J Hematol Blood Transfus.

[16] Talwar D, Kumar S, Acharya S, Khanna S, Hulkoti V (2021). Paroxysmal supraventricular tachycardia and cardiac arrest: A presentation of pulmonary embolism with infarction as a sequela of long COVID syndrome. Cureus.

